# Conjugation of ampicillin and enrofloxacin residues with bovine serum albumin and raising of polyclonal antibodies against them

**DOI:** 10.14202/vetworld.2016.410-416

**Published:** 2016-04-26

**Authors:** B. Sampath Kumar, Vasili Ashok, P. Kalyani, G. Remya Nair

**Affiliations:** Department of Veterinary Biochemistry, College of Veterinary Science, Sri Venkateswara Veterinary University, Korutla, Karimnagar, Telangana, Andhra Pradesh, India

**Keywords:** 1-ethyl-3-(3-dimethylaminopropyl) carbodiimide, antibodies against antibiotics, conjugation, indirect competitive enzyme linked immunosorbent assay

## Abstract

**Aim:**

The aim of this study is to test the potency of bovine serum albumin (BSA) conjugated ampicillin (AMP) and enrofloxacin (ENR) antigens in eliciting an immune response in rats using indirect competitive enzyme-linked immunosorbent assay (icELISA).

**Materials and Methods:**

AMP and ENR antibiotics were conjugated with BSA by carbodiimide reaction using 1-ethyl-3-(3-dimethylaminopropyl) carbodiimide (EDC) as a cross-linker. The successful conjugation was confirmed by sodium dodecyl sulfate polyacrylamide gel electrophoresis. Sprague-Dawley rats were immunized with the conjugates and blood samples were collected serially at 15 days time interval after first immunization plus first booster, second booster, third booster, and the fourth sampling was done 1½ month after the third booster. The antibody titres in the antisera of each antibiotic in all the four immunization cycles (ICs) were determined by an icELISA at various serum dilutions ranging from 1/100 to 1/6400.

**Results:**

Analysis of antibiotic-BSA conjugates by sodium dodecyl sulfate polyacrylamide gel electrophoresis and coomassie blue staining revealed high molecular weight bands of 85 kDa and 74 kDa for AMP-BSA and ENR-BSA respectively when compared to 68 kDa band of BSA. Both the antibiotic conjugates elicited a good immune response in rats but comparatively the response was more with AMP-BSA conjugate than ENR-BSA conjugate. Maximum optical density 450 value of 2.577 was recorded for AMP-BSA antisera, and 1.723 was recorded for ENR-BSA antisera at 1/100^th^ antiserum dilution in third IC.

**Conclusion:**

AMP and ENR antibiotics proved to be good immunogens when conjugated to BSA by carbodiimide reaction with EDC as crosslinker. The polyclonal antibodies produced can be employed for detecting AMP and ENR residues in milk and urine samples.

## Introduction

Antibiotics are being used extensively in the treatment of sick animals. They are also being used as growth promoters and prophylactic agents in lactating animals which is responsible for the presence of their residues in the milk [1]. Beta-lactam antibiotics like penicillin are the most frequently used, the residues of which can produce detrimental effects such as allergic reactions in humans who are sensitive to beta-lactams [2]. It may also lead to the development of antibiotic-resistant strains of bacteria [3]. Antibiotics when used inappropriately and irrationally it provides favorable conditions for the development of a resistant group of microbes that can spread very easily [4]. Enrofloxacin (ENR) is widely used in the treatment of infectious diseases because of its broad spectrum activity, and this may result in persistence of ENR residues in animal body which in turn might lead to the development of drug-resistant bacterial strains or allergies in the animal [5]. Dinki and Balcha [6] reported antibiotic residues in 28 samples with 23.3% detection rate in cattle milk samples collected from six different milk collection centres in Guwahati city in India. Gentamycin and streptomycin residues were estimated to be 90 µg/L and 80 µg/L respectively by Zeina *et al*. [7].in cattle milk in Lebanon which were below the maximum residue limit of 200 µg/L set by FAO/WHO. A study conducted in Nepal by Dhakal *et al*. [8] revealed that mastitis pathogens have developed resistance to ampicillin (AMP) and penicillin. Gentamycin and streptomycin are found to be developing resistance. All these results emphasize the need to have strict control measures on the use of antibiotics in veterinary practice both as therapeutic as well as prophylactic agents and also the need to have rapid and sensitive screening methods to detect antibiotic residues in milk.

Antibiotics are small molecules with molecular weights of <1 kDa (haptens) and to elicit an immune response, they have to be conjugated with carrier molecules such as bovine serum albumin (BSA) [9].

Antibodies are utilized for analysis, purification, and enrichment, and to mediate or modulate physiological responses. The ability of antibodies to bind an antigen with a high degree of affinity and specificity has led to their ubiquitous use in a variety of scientific and medical disciplines. Their use in diagnostic assays and as therapeutics has had a profound impact on the improvement of health and welfare in both humans and animals [10].

This study was undertaken with the objective of producing polyclonal antibodies (pAbs) against AMP and ENR antibiotics by conjugating them with BSA and detection of these pAbs by a sensitive indirect competitive enzyme linked immunosorbent assay (icELISA) in antibiotic specific antisera.

## Materials and Methods

### Ethical approval

The experimental protocol was approved by the Institutional Animal Ethics Committee under order No. 8/i/10.

### Animals

The rats (Sprague-Dawley) for the experiment were procured from the National Institute of Nutrition, Hyderabad (No. DBT/LAISC 2455). The Sprague-Dawley rats aged 7-8 weeks were kept under well lighted experimental house and maintained on standard rat feed with *ad libitum* water. A total three groups with three rats in each were maintained, two test groups (for AMP and ENR) and one control group.

### Conjugation of AMP and ENR with BSA

AMP was conjugated with BSA as per the method described by Samsonova *et al*. [11] with slight modifications whereas ENR was conjugated by the method described by Sui *et al*. [12]. For conjugation 2.5 ml of AMP (100 mg/ml) and 20 mg of BSA were taken in a clean beaker and 2.5 ml of ENR (100 mg/ml) and 20 mg of BSA were taken in another clean beaker. 580 mg of 1-ethyl-3-(3-dimethylaminopropyl) carbodiimide (EDC) was dissolved in 2 ml of distilled water and was added drop-wise to each of the above mixtures separately, accompanied by continuous stirring on a magnetic stirrer. The pH of the solutions was adjusted to 5.0-6.0 by adding 0.1 N HCl.

The above reaction mixtures of AMP-EDC-BSA and ENR-EDC-BSA were incubated at room temperature (RT) in separate beakers with continuous stirring for 2 h. After the reaction time of 2 h, uncoupled antibiotic and EDC were removed by dialysis. Dialysis membrane having the cut-off molecular weight of 12-14 kDa was procured from Hi-Media (Cat.No.DM003). Dialysis was performed according to the method described by Bollag *et al*. [13]. The samples were dialyzed against phosphate buffer saline (PBS) (pH – 7.4) with four changes, each for 8 h. The conjugated samples were analyzed by sodium dodecyl sulfate polyacrylamide gel electrophoresis (SDS-PAGE) to confirm successful conjugation [14]. SDS-PAGE was performed according to the method described by Christoph [14]. The images of the stained gels were taken in the gel documentation system (G-box-Syngene).

### Immunogen preparation

For primary immunization, AMP and ENR immunogens were prepared by adding 40 µl of each of the two conjugates separately to 460 µl PBS and 500 µl of complete Freund’s adjuvant. AMP and ENR booster immunogens were prepared by adding 40 μl of the conjugate to 460 µl of PBS buffer and 500 µl of incomplete Freund’s adjuvant as described by Dykman *et al*. [15].

The immunogen was mixed thoroughly, and 300 µl was injected to each rat (test group) subcutaneously at two different sites (150 µl at each site) according to the immunization schedule as described by Dykman *et al*. [15].

### Collection of blood from rats

The blood was collected by orbital sinus venipuncture method described by Oruganti and Gaidhani [16]. A total of four blood collections were made in each group at different time intervals according to the schedule given in Table-1.

**Table-1:** Immunization schedule.

Immunization schedule	Procedure
Day 0	1^st^ immunization antigen+CFA
Day 15	1^st^ boost antigen+ICFA
Day 30	1^st^ test bleed
Day 37	2^nd^ boost antigen+ICFA
Day 52	2^nd^ test bleed
Day 59	3^rd^ boost antigen+ICFA
Day 74	3^rd^ test bleed
Day 104	4^th^ test bleed

CFA=Complete Freund’s adjuvant, ICFA=Incomplete Freund’s adjuvant

### Estimation of total protein, albumin, and A/G ratio

The serum samples of rats from test and control groups collected after second booster (third immunization cycle [IC]) were analyzed for total protein, albumin and A/G ratio by using ensure biotech total protein and albumin teaching kit.

### Preparation of ELISA antigens (casein-antibiotic conjugates)

0.83 µmol of casein was dissolved in 2 ml of distilled water in the presence of small amount of sodium-bi-carbonate to maintain alkaline condition. 83 µmol of antibiotic and 83 µmol of EDC were added to the above protein solution. The reaction mixture was stirred on a magnetic stirrer continuously for 2 h at RT. The pH of the solution was adjusted to 5.0. Reaction mixtures of both the antibiotics were then incubated overnight at 4°C. Conjugates were dialyzed against distilled water as per the method given by Samsonova *et al*. [11].

### Standardization of icELISA

The serum samples collected after the second booster (third IC) were used for the standardization. Checkerboard titration was performed using different dilutions of antigens against different serum dilutions of test groups and negative control at constant secondary antibody-horseradish peroxidase (HRP) conjugate dilution of 1/10,000 (manufacturer’s instruction). Serial antigen dilutions (from 2 × 10^6^ ng/ml to 2 ng/ml) were taken from rows B to H in 96 well polystyrene plates and serial primary antibody dilutions (from 1/50 to 1/1600) were taken from columns 1 to 6 for test group samples and columns 7 to 12 for control group samples. The dilution of antigen which showed, maximum absorbance reading and started to maintain almost a stationary phase was taken as the optimum according to the procedure described by Fan *et al*. [17].

### Indirect ELISA

96 well flat bottom polystyrene ELISA plates (Nunc, Denmark) were coated with 250 µl of antigen (antibiotic-casein conjugate) in 0.01 M carbonate buffer (pH - 9.6). The plates were incubated overnight at 4°C. The wells were washed 3 times with 250 µl/well of PBS that contained 0.05% tween 20 (PBST). The free (unbound) sites were blocked with 250 µl/well of blocking buffer containing 2% casein. The plates were incubated at 37°C for 1 h. The wells were washed 3 times with PBS, 250 µl/well. 100 µl of antiserum samples with two-fold dilutions from 1/100 to 1/6400 in PBST were added to each well and the plate was incubated for 1 h at 37°C. The wells were washed 3 times with PBST (250 µl/well per wash cycle). 100 µl of a conjugate of secondary antibodies with HRP in PBST was added to each well and the plate was incubated for 1 h at 37°C. The wells were washed 3 times with PBST (250 µl/well per wash cycle). 100 µl of the substrate (3, 3’, 5, 5’ tetramethylbenzidine) in PBST (1 in 20 dilution) was added to each well. The reaction was stopped after 10-15 min by adding 50 µl/well of 4M H_2_SO_4_ as the method described by Samsonova *et al*. [11]. Optical density (OD) was measured by using ELISA microtitre plate reader at 450 nm (Biotech instrument-µquant).

### Testing the antiserum samples for antibody titres

The antibody titres in the serum samples collected from immunized rats were tested by icELISA standardized as described above. The optimum antigen concentrations and primary antibody dilutions obtained for all the four antibiotics by the above-described method were used for the test. The antisera of all the three animals in each group collected during all the three ICs and 4^th^ sampling were tested at various serum dilutions ranging from 1/100 to 1/1600. Each sample was tested in duplicate including the control serum samples. In the reagent blank, PBST was added instead of antiserum. In the negative control wells, serum samples of control group rats were added. The mean OD_450_ of various serum dilutions at each IC for each group of rats were used to plot a graph with absorbance on Y-axis and serum dilutions on X-axis.

### Construction of PNT baseline

The mean and the standard deviation (SD) values of the control group at each dilution ranging from 1/100 to 1/6400 were calculated for each of the two different antigen coated plates used in this study. Three units of SD was added to the corresponding mean absorbance value and a graph was plotted with values of mean plus 3 times SD (M + 3SD) on Y-axis and serum dilutions on X-axis. This was considered as positive, negative threshold (PNT) baseline [18]. Separate PNT baselines were constructed for each test group.

### Prediction of antibody titres

The positive antibody titres were determined based on the cut-off value obtained from PNT baseline constructed. The highest OD_450_ value of the PNT baseline rounded off to the nearest single digit decimal was taken as cut-off value. The OD_450_ value over and above the cut-off value was considered positive antibody titer [18].

## Results and Discussion

### Determination of successful conjugation

**Figure 1:**
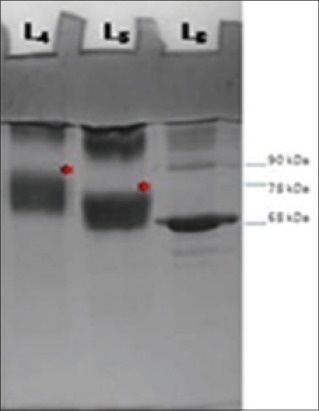
Coomassie blue stained sodium dodecyl sulfate polyacrylamide gel electrophoresis gel of antibiotic conjugates: L_4_: Ampillicin conjugate, L_5_: Enrofloxacin conjugate, L_6_: Bovine serum albumin (*) conjugates.

Analysis of antibiotic conjugates by SDS-PAGE and coomassie blue staining revealed higher molecular weights of antibiotic-BSA conjugates when compared to normal BSA (Figure-1). Before conjugation with antibiotic, the molecular weight of BSA was 68 KDa. After conjugation, the molecular weights of conjugates were 85 kDa, 74 kDa for AMP-BSA and ENR-BSA, respectively. These results clearly indicate the successful conjugation of antibiotics with BSA and are similar to those obtained by Jiang *et al*. [19] who analyzed sarafloxacin-BSA conjugate by SDS-PAGE and observed an increase in the molecular weight of the conjugate when compared to the carrier protein BSA.

### Estimation of total protein, albumin and A/G ratio

The mean values of total protein, albumin, globulin, and A/G ratio of serum samples collected during third IC. The mean total protein concentration was 12±1.15 g/dL and 24±1.73 g/dL for AMP and ENR antisera, respectively and in the control group, it was 6.66±0.01 g/dL. The mean albumin concentration was 3.36±0.173 g/dL, 3.21±0.003 g/dL for AMP and ENR antisera are respectively compared to 3.36±0.173 g/dL in the control group. The mean globulin concentration was 8.64±0.10 g/dL and 20.79±0.08 g/dL for AMP and ENR antisera, respectively, whereas it was .30±0.04 g/dL in the control group. The mean A/G ratio was 0.38±0.008 and 0.15±0.017 for AMP and ENR antisera, respectively. The mean A/G ratio in the control group serum was 1.02±0.012. Increased globulin concentration and decreased A/G ratio indicates the presence of antibodies in the antisera.

### Standardization of antigen concentration and antiserum dilution for indirect ELISA

The checker board titration results are presented in Table-2 for AMP-casein conjugate and in Table-3 for ENR-casein conjugate. In all the titrations, the OD_450_ values of the test wells increased suddenly from the coated antigen concentration of 2 to 20 ng/ml and continued to maintain a steady phase at higher concentrations. So, 20 ng/ml was chosen as optimum antigen concentration. The absorbance values of the negative control wells dropped suddenly from serum dilution of 1/50 to 1/100 and continued to maintain similar range at higher dilutions and at all antigen concentrations. The test wells continued to maintain higher absorbance values at corresponding dilutions. Hence, 1/100 was chosen as optimum serum dilution. The highest M + 3SD values obtained for negative sera were 0.213 (Table-4) and 0.209 (Table-5) for AMP-casein and ENR-casein coated plates, respectively. Hence, the cut-off value was selected as 0.3 for antibiotics.

**Table-2:** Checker board titration to standardize optimum casein-AMP conjugate and antiserum dilution.

Concentration of Well antigen (ng/ml)		Test serum sample						Negative control					
	
		Dilutions of antisera						Dilutions of antisera					
	
		1/50	1/100	1/200	1/400	1/800	1/1600	1/50	1/100	1/200	1/400	1/800	1/1600
	
		1	2	3	4	5	6	7	8	9	10	11	12
Blank	A	0.148	0.159	0.078	0.190	0.111	0.178	0.091	0.163	0.143	0.155	0.140	0.147
2×10^6^	B	2.983	2.545	2.068	1.976	1.587	1.057	0.624	0.192	0.187	0.189	0.195	0.116
2×10^5^	C	2.745	2.451	1.987	1.852	1.456	0.958	0.525	0.112	0.185	0.165	0.159	0.112
2×10^4^	D	2.415	2.316	1.886	1.689	1.321	0.845	0.656	0.184	0.158	0.195	0.188	0.159
2×10^3^	E	2.221	2.215	1.769	1.521	1.205	0.833	0.587	0.156	0.165	0.189	0.125	0.141
200	F	2.115	2.110	1.645	1.361	1.124	0.782	0.495	0.121	0.198	0.156	0.151	0.128
20	G	2.021	1.994	1.607	1.246	0.972	0.759	0.458	0.184	0.185	0.136	0.102	0.196
2	H	1.498	1.331	1.026	0.752	0.578	0.396	0.321	0.146	0.175	0.117	0.085	0.129

AMP=Ampicillin

**Table-3:** Checker board titration to standardize optimum caseinENR conjugate and antiserum dilution.

Concentration of Well antigen (ng/ml)		Test serum sample						Negative control					
	
		Dilutions of antisera						Dilutions of antisera					
	
		1/50	1/100	1/200	1/400	1/800	1/1600	1/50	1/100	1/200	1/400	1/800	1/1600
	
		1	2	3	4	5	6	7	8	9	10	11	12
Blank	A	0.109	0.103	0.100	0.088	0.125	0.109	0.134	0.126	0.154	0.163	0.128	0.133
2×10^6^	B	2.598	2.326	2.159	1.998	1.652	1.450	0.556	0.116	0.185	0.156	0.149	0.112
2×10^5^	C	2.441	2.236	2.079	1.789	1.536	1.241	0.531	0.121	0.186	0.152	0.145	0.198
2×10^4^	D	2.326	2.187	1.969	1.656	1.324	1.105	0.401	0.178	0.156	0.123	0.189	0.119
2×10^3^	E	2.158	2.056	1.851	1.523	1.121	0.959	0.459	0.189	0.187	0.125	0.197	0.157
200	F	2.103	1.925	1.754	1.386	0.956	0.843	0.358	0.142	0.195	0.178	0.185	0.139
20	G	2.044	1.889	1.595	1.027	0.819	0.775	0.338	0.196	0.183	0.138	0.118	0.141
2	H	1.156	0.721	0.521	0.495	0.352	0.229	0.352	0.185	0.189	0.119	0.158	0.176

ENR=Enrofloxacin

**Table-4:** Mean OD_450_ values of indirect ELISA of AMP antisera.

ICs	Serum dilutions						

	1/100	1/200	1/400	1/800	1/1600	1/3200	1/6400
1^st^ IC	1.499	1.156	0.840	**0.668**	0.458	0.323	0.281
2^nd^ IC	1.781	1.607	1.429	1.225	**0.924**	0.568	0.436
3^rd^ IC	2.577	2.195	1.841	1.619	1.453	1.417	**1.184**
4^th^ collection	1.944	1.601	1.267	**0.932**	0.756	0.642	0.558
Negative control (M+3SD)	0.213	0.199	0.157	0.12	0.107	0.105	0.107

Bold numbers indicate 50% titres. IC=Immunization cycle, OD=Optical density, ELISA=Enzyme linked immunosorbent assay, AMP=Ampicillin

**Table-5:** Mean OD_450_ values of indirect ELISA of ENR antisera.

IC	Serum dilutions						
	1/100	1/200	1/400	1/800	1/1600	1/3200	1/6400
1^st^ IC	1.018	0.735	**0.447**	0.297	0.148	0.099	0.065
2^nd^ IC	1.252	1.046	0.759	**0.628**	0.445	0.302	0.272
3^rd^ IC	1.723	1.476	1.241	1.091	0.94	**0.893**	0.755
4^th^ Sampling	1.04	0.783	**0.565**	0.461	0.399	0.275	0.191
Negative control (M+3SD)	0.209	0.216	0.158	0.16	0.115	0.105	0.099

Bold numbers indicate 50% titres. IC=Immunization cycle, OD=Optical density, ELISA=Enzyme linked immunosorbent assay, ENR=Enrofloxacin, SD=Standard deviation

### Detection of antibody titres in the AMP antisera

**Figure 2:**
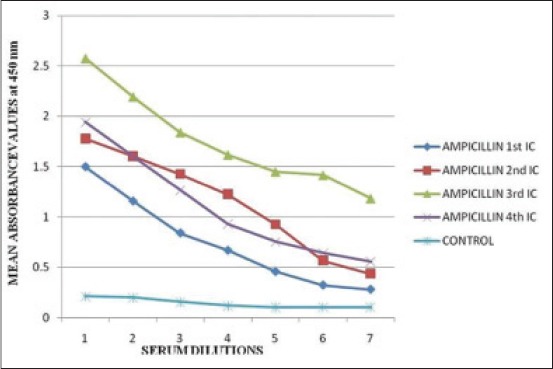
Calibration curve for indirect enzyme linked immunosorbent assay of bovine serum albumin-ampicillin antisera at various immunization cycles (1, 2, 3, 4, 5, 6, 7 represents serum dilutions of 1/100, 1/200, 1/400, 4/800, 1/1600, 1/3200, and 1/6400 respectively).

**Figure 3:**
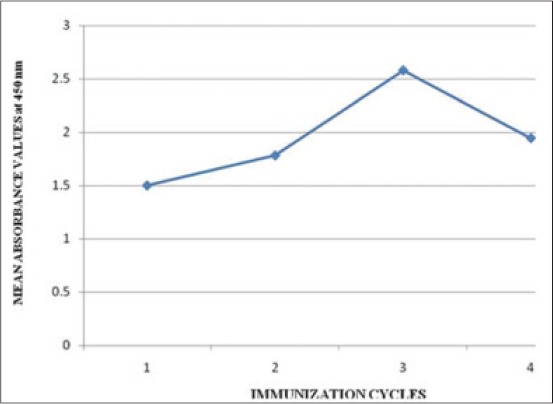
Calibration curve for indirect enzyme linked immunosorbent assay of bovine serum albumin-ampicillin antisera at 1/100^th^ serum dilution of various immunization cycles (ICs) (1, 2, 3, 4 represents serum dilutions of 1^st^, 2^nd^, 3^rd^, 4^th^ ICs respectively at 1/100^th^ serum dilation).

The mean of the corrected OD values of the three animals in AMP test group and M + 3SD of the three animals in each control group at various serum dilutions and various ICs are depicted in Table-4. The highest M + 3SD value was 0.213. The cut-off value was selected as 0.3 (nearest single digit decimal above 0.213). The mean OD_450_ values of the serum samples from immunized rats were above the cut-off value up to serum dilution of 1/3200 in all the three ICs and fourth collection which indicated positive antibody titers (Figure-2). The values of 50% antibody titres increased from the antiserum dilution of 1/800 in first IC to 1/6400 in third IC as depicted in Table-4. The AMP antisera gave positive antibody titers up to a dilution of 1/3200 in first IC and 1/6400 in second and third ICs and fourth sampling. Maximum OD_450_ value of 2.577 was obtained at 1/100 antiserum dilution in third IC which clearly indicated that the immune response was the highest in third IC. The immune response significantly increased from first IC to third IC at 1/100 antiserum dilution and decreased in fourth collection (Figure-3). Anti-AMP antibodies were successfully produced by Strasser *et al*. [20] in rabbits which were confirmed by double antibody solid phase ELISA. The maximum antibody titer of 1:550,000 was obtained after third booster injection (fourth IC) at working antiserum dilution of 1:1000 which is much higher than 1/6400 in this study. The difference could be attributed to species variations in the ability to produce antibodies. Competitive ELISA used by McConnell *et al*. [21] for AMP antisera yielded positive antibody titers indicating clearly that AMP-carrier protein conjugate was immunopotent and capable of eliciting specific immune response.

### Detection of antibody titres in the ENR antisera

**Figure 4:**
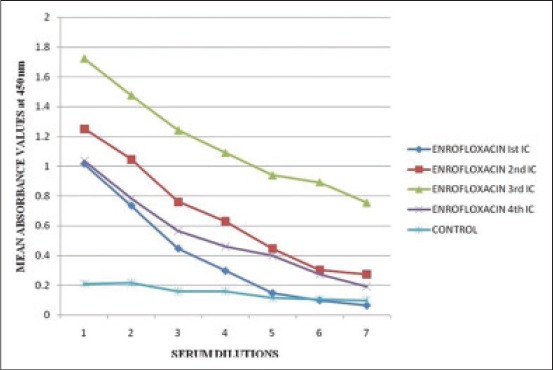
Calibration curve for indirect enzyme linked immunosorbent assay of bovine serum albumin-enrofloxacin antisera at various immunization cycles (1, 2, 3, 4, 5, 6, 7 represents serum dilutions of 1/100, 1/200, 1/400, 4/800, 1/1600, 1/3200, and 1/6400 respectively).

**Figure 5:**
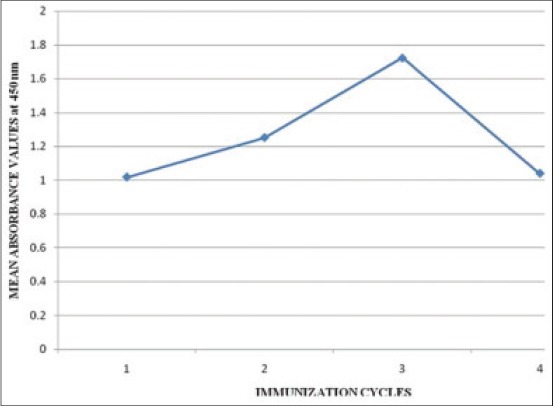
Calibration curve for indirect enzyme linked immunosorbent assay of bovine serum albumin-enrofloxacin antisera at 1/100^th^ serum dilution of various immunization cycles (IC) (1, 2, 3, 4 represents serum dilutions of 1^st^, 2^nd^, 3^rd^, 4^th^ ICs respectively at 1/100^th^ serum dilation).

The results of corrected mean OD_450_ values of the test group (three animals) and M + 3SD values of the control group (three animals) at various serum dilutions and various ICs are depicted in Table-5. The highest M + 3SD value was 0.209. The cut-off value was selected as 0.3 (nearest single digit decimal above 0.209). The mean OD_450_ values of the ENR antisera were above the cut-off value up to a dilution of 1/400 in first IC and up to dilution of 1/1600 in second and third and ICs and fourtth collection which indicated positive antibody titres (Figure-4). The immune response significantly increased from first IC to third IC at 1/100 antiserum dilution and decreased in fourth sampling (Figure-5). The values of 50% antibody titres increased from the antiserum dilution of 1/400 in first IC to 1/3200 in third IC as depicted in Table-5. The ENR antisera gave positive antibody titres up to a dilution of 1/400 in first IC, 1/3200 in second IC, 1/6400 in third IC and 1/1600 in fourth sampling. Maximum OD_450_ value of 1.723 was obtained at 1/100 antiserum dilution in third IC which clearly indicated that the immune response was the highest in third IC. Liu *et al*. [22] used ELISA and competitive inhibition ELISA to determine antibody titers in ENR antisera and obtained antibody titre as high as 1:250,000 for three analogs of ENR belonging to fluoroquinolone family.

### Comparative immunogenic potency of AMP-BSA and ENR-BSA antisera

**Figure 6:**
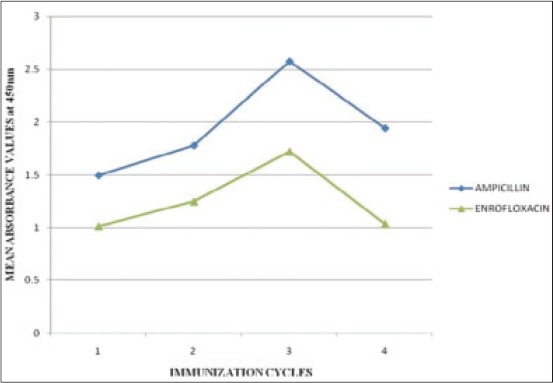
Comparative calibration curves for indirect enzyme linked immunosorbent assay of bovine serum albumin-antibiotic antisera at 1/100^th^ serum dilution of various immunization cycles (ICs) (1, 2, 3, 4 represents serum dilutions of 1^st^, 2^nd^, 3^rd^, 4^th^ ICs respectively at 1/100^th^ serum dilation).

Highest immune response was seen in AMP antiserum followed by ENR evidenced by OD_450_ values of 2.577, 1.723 for AMP and ENR antisera respectively at 1/100 serum dilution in third IC (Figure-6).

### Raised antibodies to detect the AMP and ENR in milk samples

To use produces antibodies for the detection of antibiotics in milk samples, we need large quantities of antibodies for which the work has to be scaled up and large number of animals have to be maintained to obtain sufficient antiserum for harvesting antibodies. This is the first part of work done to test the efficacy of BSA conjugates of AMP and ENR in eliciting immune response in rats. The second part of work is to scale up the process and use the antibodies in developing quantitative assay or lateral flow through assay to detect antibiotic residues in milk samples.

### Novelty and contribution to the animal health

This work mostly concerned with the public health importance. Antibiotics residues in milk higher than maximum residue levels are of great concern to dairy farmers, milk processors, regulatory agencies, and consumers due to their possible adverse effects on people allergic to antibiotics, potential buildup of antibiotic-resistant organisms in humans [23]. The novelty of this work lies in using BSA as a suitable conjugating agent with EDC as coupler for AMP and ENR which are otherwise haptens and hence incapable of eliciting immune response.

## Conclusion

In this study, the antibiotics (AMP and ENR) were successfully conjugated with carrier protein BSA by carbodiimide reaction using EDC as a cross linker. These conjugated antibiotics were capable of producing pAbs which was confirmed by indirect ELISA.

## Authors’ Contributions

BSK and PK collected the blood samples from rats for the study. BSK carried out the current investigation under the guidance of VA. BSK drafted and revised the manuscript under the guidance of PK. GRN helped in carry out the ELISA part of work. All the authors have read and approved the manuscript.

## References

[1] Singh S, Shukla S, Tandia N, Kumar N, Paliwal R (2014). Antibiotic residues: A global challenge. Pharm. Sci. Monitor.

[2] Fitzgerald S.P, Loan N, Connell R.M, Benchi E.KH, Kane N (2007). stable competitive Enzyme-Linked immunosorbent assay kit for rapid measurement of 11 active beta-lactams in milk, tissue, urine and serum. J. AOAC. Int.

[3] Kebede G, Zenebe T, Disassa H, Tolosa T (2014). Review on detection of antimicrobial residues in raw bulk milk in dairy farms. AJBAS.

[4] Padol A.R, Malapure C.D, Domple V.D, Kamdi B.P (2015). Occurance, public health implications and detection of antibacterial drug residues in cow milk. Environ. We Int. J. Sci. Tech.

[5] Yan H, Wang H, Qin X, Liu B, Du J (2011). Ultra sound-assisted dispersive liquid-liquid micro extraction for determination of fluroquinolones in pharmaceutical waste water. J. Pharm. Biomed. Anal.

[6] Dinki N, Balcha E (2013). Detection of antibiotic residues and determination of microbial quality of raw milk from milk collection centres. Adv. Anim. Vet. Sci.

[7] Zeina K, Pamela A.K, Fawwak S (2013). Quantification of antibiotic residues and determination of antimicrobial resistance profiles of microorganisms isolated from bovine milk in Lebanon. Food Nutr. Sci.

[8] Dhakal I.P, Dhakal P, Koshihara T, Nagahata H (2007). Epidemiological and bacteriological survey of buffalo mastitis in Nepal. J. Vet. Med. Sci.

[9] Suzanne N.S, Lioyd E.M (2003). In: Food Analysis.

[10] Lipman N.S, Jackson L.R, Trudel L.J, Weis-Garcia F (2005). Monoclonal versus polyclonal antibodies: Distinguishing characteristics, applications, and information resources. ILAR J.

[11] Samsonova Z.V, Shchelokova O.S, Ivanova N.L, Rubtsova M.Y, Egorov A.M (2005). Enzyme-linked immunosorbent assay of ampicillin in milk. J. Appl. Biochem. Microbiol.

[12] Sui J, Hong L, Limin C, Zhenxing L (2009). Dot-immunogold filtration assay for rapid screening of three fluroquinolones. J. Food Agric. Immunol.

[13] Bollag D, Micheal D.R, Stuart J.E (1996). In: Protein Methods.

[14] Christoph K (2002). Posttranslational modifications of proteins: tools for proteomics. In: methods in molecular biologypp.

[15] Dykman L.A, Sumaroka M.V, Staroverou S.A, Zaltseva I.S, Bogatyrev V.A (2004). Immunogenic properties of colloidal gold. Biol. Bull.

[16] Oruganti M, Gaidhani S (2011). Routine bleeding techniques in laboratory rodents. IJPSR.

[17] Fan G.Y, Yang R.S, Jiang J.Q, Chang X.Y, Chen J.J, Qi Y.H, Wu S.X, Yang X.F (2012). Development of a class-specific polyclonal antibody based indirect competitive ELISA for detecting fluoroquinolone residues in milk. J. Zhejiang Univ. Sci. B.

[18] Ramadass P, Parthiban M, Thiagarajan V, Chandrasekar M, Vidhya M, Raj G.D (2008). Development of single serum dilution ELISA for detection of infectious bursal disease virus antibodies. J. Vet. Arch.

[19] Jiang J, Zhang H, Wang Z (2011). Development of an immunoassay for determination of fluroquinolones pollutant in environmental water sample. International conference on nanotechnology and biosensors. IPCBEE.

[20] Strasser A, Usleber E, Schneider E, Dietrich R, Burk C, Martlbauer E (2003). Improved enzyme immune assay for group-specific determination of penicillins in milk. J. Food Agric. Immunol.

[21] McConnell R.I, Elouard B, Stephen P.F, John V.L (2003). Method and kit for detecting, or determining the quantity of beta-lactam penicillins. United States Patent, Patent No. US 6960653 B2.

[22] Liu C, Hong L, Limin C, Jie J (2005). Anti-ENR antibody production by using ENR-screened HSA as an immunogen. J. Ocean U China.

[23] Tiwari R, Chakraborty S, Dharma K, Rajagunalan S, Singh S.V (2013). Antibiotic resistance - An emerging health problem: Causes, worries, challenges and solutions –A review. Int. J. Curr. Res.

